# Changes in Body Size during Early Growth Are Independently Associated with Arterial Properties in Early Childhood

**DOI:** 10.3390/jcdd8020020

**Published:** 2021-02-17

**Authors:** Juan M. Castro, Mariana Marin, Agustina Zinoveev, Victoria García-Espinosa, Pedro Chiesa, Daniel Bia, Yanina Zócalo

**Affiliations:** 1 Departamento de Fisiología, Facultad de Medicina, Centro Universitario de Investigación, Innovación y Diagnóstico Arterial (CUiiDARTE), Universidad de la República, General Flores 2125, 11800 Montevideo, Uruguay; jmcastrobrugnoli@gmail.com (J.M.C.); mmarin5213@gmail.com (M.M.); a.zinoveev@gmail.com (A.Z.); victoria.s.garcia.espinosa@gmail.com (V.G.-E.); 2 Servicio de Cardiología Pediátrica, Centro Hospitalario Pereira-Rossell, ASSE-Facultad de Medicina, Universidad de la República, Bulevar Artigas 1550, 11600 Montevideo, Uruguay; chiesacorradospd@gmail.com

**Keywords:** aortic pressure, arterial stiffness, birth weight, blood pressure, body size trajectories, children, intima–media thickness

## Abstract

Nutritional status in early life stages has been associated with arterial parameters in childhood. However, it is still controversial whether changes in standardized body weight (z-BW), height (z-BH), BW for height (z-BWH) and/or body mass index (z-BMI) in the first three years of life are independently associated with variations in arterial structure, stiffness and hemodynamics in early childhood. In addition, it is unknown if the strength of the associations vary depending on the growth period, nutritional characteristics and/or arterial parameters analyzed. Aims: First, to compare the strength of association between body size changes (Δz-BW, Δz-BH, Δz-BWH, Δz-BMI) in different time intervals (growth periods: 0–6, 0–12, 0–24, 0–36, 12–24, 12–36, 24–36 months (m)) and variations in arterial structure, stiffness and hemodynamics at age 6 years. Second, to determine whether the associations depend on exposure to cardiovascular risk factors, body size at birth and/or on body size at the time of the evaluation (cofactors). Anthropometric (at birth, 6, 12, 24, 36 m and at age 6 years), hemodynamic (peripheral and central (aortic)) and arterial (elastic (carotid) and muscular (femoral) arteries; both hemi-bodies) parameters were assessed in a child cohort (6 years; *n* =632). The association between arterial parameters and body size changes (Δz-BW, Δz-BH, Δz-BWH, Δz-BMI) in the different growth periods was compared, before and after adjustment by cofactors. Results: Δz-BW 0–24 m and Δz-BWH 0–24 m allowed us to explain inter-individual variations in structural arterial properties at age 6 years, with independence of cofactors. When the third year of life was included in the analysis (0–36, 12–36, 24–36 m), Δz-BW explained hemodynamic (peripheral and central) variations at age 6 years. Δz-BH and Δz-BMI showed limited associations with arterial properties. Conclusion: Δz-BW and Δz-BWH are the anthropometric variables with the greatest association with arterial structure and hemodynamics in early childhood, with independence of cofactors.

## 1. Introduction

Changes in body size (e.g., in body weight or height z-scores (Δz-BW, Δz-BH)) during childhood growth have been associated with arterial changes and cardiovascular (CV) risk [1,2,3,4,5,6,7,8]. In particular, a rapid or extreme growth in early life has been associated with increased CV risk in adult life. However, those findings have not been universal. Differences in (i) the anthropometric parameters analyzed (e.g., z-BW, z-BH, z-BW for height (z-BWH) and/or z-body mass index (z-BMI)), (ii) the arterial properties studied (e.g., structural vs. functional; local vs. regional; central vs. peripheral), (iii) subjects’ characteristics (e.g., age, race) and/or (iv) the methodological approaches considered (e.g., growth periods) contribute to explaining the controversies and differences among available data. Early growth-related anthropometric changes would be associated with the development of or exposure to factors related to increased CV risk (e.g., hypertension, obesity). Then, the controversial findings and lack of a clear understanding could also be related to the inherent complexity given by dependence on prior anthropometric conditions and/or cofactors. In this context, the following questions are to be answered:

(1) Are early variations in z-BW, z-BH, z-BWH and/or z-BMI associated with arterial variations at age 6 years (y.), with independence of exposure to CV risk factors (CRFs), body size at birth and/or body size at the time of the study?
(2) Which is the anthropometric index whose changes show significant strength of association with arterial parameter variations at age 6 years?
(3) Do the associations depend on the growth interval considered?
(4) Are early changes in body size mainly associated with hemodynamic (e.g., systolic (SBP), diastolic (DBP) or pulse pressure (PP), wave-derived parameters), structural (e.g., diameter, intima–media thickness) or stiffness parameters?
(5) Are they primarily associated with peripheral (pBP) or with central pressure (cBP)?

Regarding the first two questions, it is noted that subjects with high rates of growth-related body size increase show high levels of exposure to CRFs [9,10,11,12,13], which makes it difficult to identify whether (potential) arterial variations can be explained (directly and/or indirectly) by growth rates. Available data mainly came from studies done in adolescents or adults and in premature or small for gestational age subjects. Therefore, the growth impact on arterial parameters in (healthy) children is still unknown. On the other hand, as stated above, works have considered different anthropometric data (e.g., z-BW [1,2,3,6], z-BH [3,6], z-BMI [7], z-BWH [5]), in many cases without an explanation of the criteria used for the selection. It is not clear whether the association (or lack thereof) between body size and arterial parameter variations depends on the studied parameter. In our knowledge, no work has analyzed and compared the main parameters used to define the anthropometric conditions in infancy and/or early childhood. Regarding the third question, different time intervals have been considered to analyze body size variations [1,2,5,6,7]. Infancy and early childhood have been recognized as critical periods in subjects’ growth and development. It is unknown whether there is a time interval in infancy and early childhood that is particularly associated with arterial parameters later in childhood (i.e., at the beginning of schoolage, at age 6 years). A“hierarchical order” has been proposed among functional and structural arterial variations associated with children’s and adolescents’ nutritional status, with pBP (rather than cBP) being the variable with the greatest variations associated with z-BMI. Aortic, but not femoral or carotid, stiffness was associated with z-BMI. In turn, arterial diameter variations were associated with z-BMI, without differences between elastic and muscular arteries [4]. Then, at least in theory, growth-related anthropometric variations could show a “hierarchical or preferential order” regarding the association with arterial parameters. With few exceptions (e.g., studies that evaluated carotid arteries in children) [5,14], available works have only evaluated the impact on pBP levels or hypertension (HT) prevalence [1,2,6,7]. In children, pBP could be elevated without a parallel increase in cBP or concomitant arterial structure or stiffness changes [15,16,17,18]. The opposite is also true. Considering the statements above, an adequate comprehensive evaluation of the association between growth-related body size and arterial changes necessarily requires evaluating complementary parameters [4].

This work’s aims were: (1) to compare the strength of association between body size changes (Δz-BW, Δz-BH, Δz-BWH, Δz-BMI) in different time intervals (growth periods: 0–6, 0–12, 0–24, 0–36, 12–24, 12–36, 24–36 m.) and variations in arterial structure, stiffness and hemodynamics at age 6 years; (2) to determine whether the associations depend on exposure to CRFs, body size at birth and/or on body size at the time of the evaluation.

## 2. Materials and Methods

### 2.1. Study Population

The study was carried out in the context of the Project Centro Universitario de Investigación, Innovación y Diagnóstico Arterial (CUiiDARTE) [4,19,20]. The protocol was approved by the Institutional Ethics Committee (Comité de Ética en Investigacón, Centro Hospitalario Pereira Rossell (Ethical approval: 29112013/29122015). Parents’ written consent and children’s assent were obtained prior to the evaluation. The cohort (*n* = 632) [4] was defined based on probabilistic, bi-stage and stratified sampling of subjects attending public kindergartens in Montevideo. It represents a sub-sample of the longitudinal study “Patrón de crecimiento, estado nutricional y calidad de alimentación en la primera infancia: análisis de su impacto sobre la estructura y función vascular y el riesgo cardiovascular relativo en niños uruguayos” (CUiiDARTE-Agencia Nacional de Investigación e Innovación (ANII), Ministerio de Desarrollo Social (MIDES), United Nations Children’s Fund (UNICEF), which started in 2010 and had a second phase in 2016. A clinical and anthropometric evaluation was carried out in each participant. In addition, data (questionnaires) on lifestyle, family and personal history were collected before non-invasive arterial evaluation.

### 2.2. Anthropometric Evaluation

Anthropometric data (BW and BH) for ages 0–36 m were obtained from health control records (mandatory at those ages according to Health Ministry regulations) and/or from self-reports documented during the interviews with parents [4]. BW and BH were measured with the participants wearing light clothing and no shoes. Standing BH was measured using a portable stadiometer and recorded to the nearest 0.1 cm. BW was measured with an electronic scale (841/843, Seca Inc., Hamburg, Germany; model HBF-514C, Omron Inc., Chicago, IL, USA) and recorded to the nearest 0.1 kg. Two measurements were always obtained and a third one was considered in case the first two readings differed by more than 0.5 cm or 0.5 kg.

Jointly considering measurements from our technicians and information from the health controls, we obtained BW and BH data corresponding to age 0 (birth), 6, 12, 24, 36 m, and 6 years. BMI was calculated as the BW to squared BH ratio. Standardized z-scores (i.e., z-BW, z-BH, z-BMI and z-BWH) were obtained (separately for males and females) by using World Health Organization software (Anthro-v.3.2.2; Anthro-Plus-v.1.0.4). z-BMI was also calculated at the time of the arterial evaluation (6 years). Changes (Δ) in standardized anthropometric indexes were determined for different time intervals (growth periods): 0–6, 0–12, 0–24, 0–36, 12–24, 12–36 and 24–36 m. The changes were always obtained by subtracting the first record from the last one (e.g., Δz-BW 0–6 m = z-BW 6 m–z-BW 0 m) [4].

### 2.3. Clinical Evaluation

None of the included subjects were taking medications, or had congenital, chronic or infectious diseases at the time of the arterial evaluation. A brief clinical interview, together with the anthropometric evaluation enabled us to assess CRF exposure. Hypertension (HT), dyslipidemia and diabetes were considered present if they had been previously diagnosed, in agreement with reference guidelines [4]. Subjects who had brachial systolic and/or diastolic pBP (pSBP and pDBP) >95th percentile for sex, age and BH during the study were considered to have high BP levels (HBP) [21]. Obesity was defined as z-BMI ≥2. A family history of CV disease (CVD) was defined by the presence of first-degree relatives with premature (<55 years in males; <65 years in females) CVD.

### 2.4. Arterial and Hemodynamic Evaluation

The evaluations were performed at the study institutions. Recordings were done after at least 10 min of rest in the supine position in a quiet, temperature-controlled room, which enabled reaching steady hemodynamic conditions.

### 2.5. Peripheral and Central Pressure and Aortic Wave-Derived Parameters

Heart rate (HR), pSBP and pDBP were obtained at 5 min intervals (Hem-4030, OmronInc., Illinois, USA). Peripheral pulse pressure (pPP = pSBP − pDBP) and mean BP (MBP = pDBP + pPP/3) were calculated. Central (aortic) BP levels (systolic (cSBP), diastolic (cDBP) and pulse (cPP)), together with wave-derived parameters were obtained using a generalized transfer function applied to peripheral (radial) BP wave records (applanation tonometry, SphygmoCor-CvMS, ATCOR Medical, Sydney, Australia). pDBP and MBP were used for calibration [4,22]. Only adequate waveforms (visual inspection) and high-quality recordings (operator index ≥85) were considered.

By means of pulse wave analysis (PWA) the first (P1) and second (P2) peaks in the cBP wave were identified. Then, their height (amplitude) and time were determined. The difference between P2 and P1 was computed as central augmented pressure (AP), and used to quantify the central aortic augmentation index (AIx = AP/cPP). Since AIx depends on HR, AIx adjusted to 75 beats/min (AIx@75) was calculated [22]. AIx is a measure of reflections’ contribution to cBP amplitude. It depends on the timing and magnitude of the reflected (backward) wave and is influenced by the compliance and structure of vessels distal to the recording site, as well as by the distance to the reflection sites. Forward and backward (Pf and Pb) components of the aortic wave were determined. Greater Pb and/or AIx values indicate increased reflections and/or earlier return of reflected waves due to increased arterial stiffness and/or closer reflection sites.

Systemic vascular resistances (SVRs), cardiac output (CO) and index (CI) were quantified from brachial pulse contour analysis (Mobil-O-Graph, I.E.M.GmbH, Stolberg, Germany) [23]. Only high-quality records (index ≤2) and satisfactory waves (visual inspection) were considered. Subjects’ values are the average of at least six consecutive records obtained in a single visit.

### 2.6. Arterial Beat-to-Beat Diameter and Intima–Media Thickness

**Figure 1:**
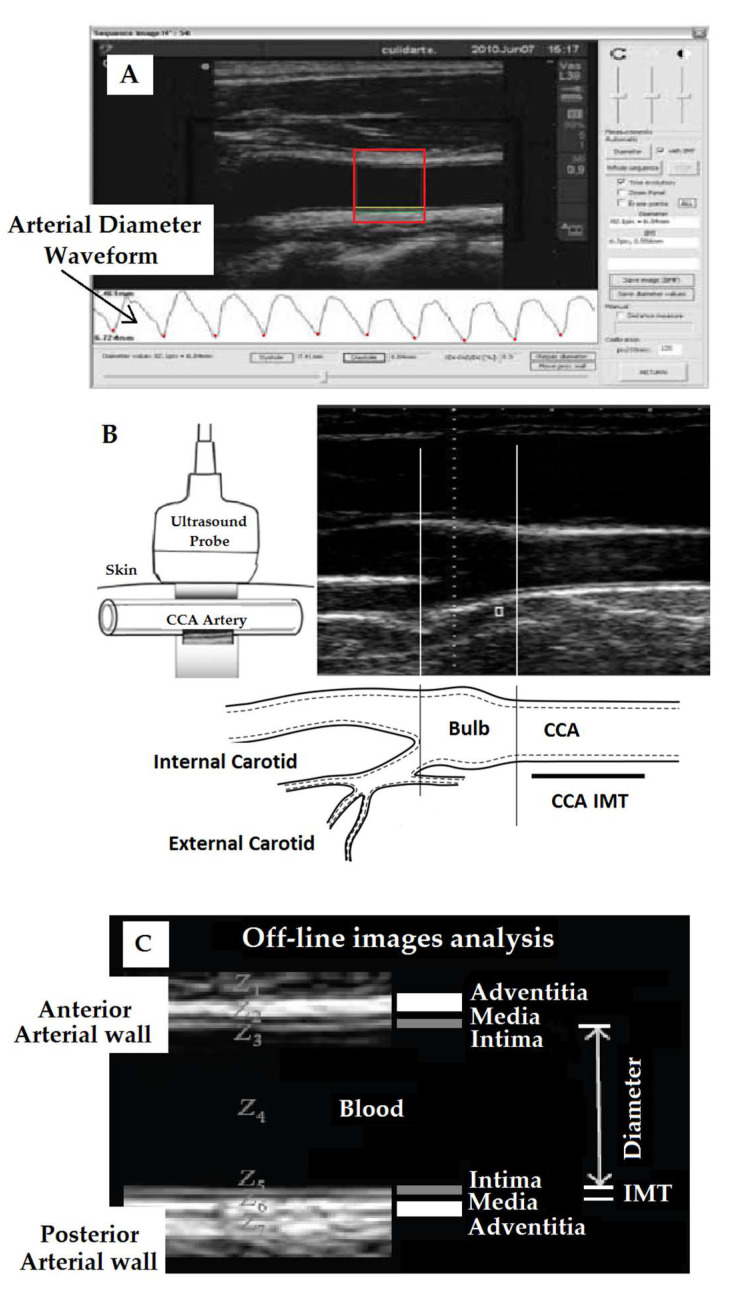
(**A**): Software for intima–media thickness (IMT) and diameter measurement (Hemodyn-4M). Red box: region of interest (ROI) to quantify diameter and IMT. (**B**,**C**): Methodological approach used to assess common carotid (CCA) and femoral artery (CFA) diameter and intima–media thickness (IMT). Z: acoustic impedance. IMT is defined as a double-line pattern visualized by echo 2D on both arterial walls in longitudinal views. Two parallel lines (leading edges of anatomical boundaries) form lumen–intima and media–adventitia interfaces. Modified from [4].

Left and right common carotid and femoral arteries (CCA, CFA) were analyzed using ultrasound (6–13MHz, M-Turbo, Sonosite Inc, Bothell, WA, USA). Sequences of images (30 s, B-Mode, longitudinal views) were stored for off-line analysis. Beat-to-beat diameter waves were obtained using border detection software (Hemodyn-4M, Dinap s.r.l., Buenos Aires, Argentina) (Figure 1A). Systolic and end-diastolic diameters (SystD and DD) and intima–media thickness (IMT, far wall, end diastole) values were obtained by averaging at least 20 beats. CCA diameter and IMT were measured 1 cm proximal to the bulb; CFA diameter and IMT were measured in a straight segment in the penultimate centimeter proximal to the bifurcation [4] (Figure 1B). IMT is the distance (mm) between the luminal border of the intima layer and the outer border of the media layer (ultrasonography). Therefore, IMT is a measurement of the thickness of the two innermost layers of the arterial wall (intima and media) (Figure 1C).

### 2.7. Local and Regional Arterial Stiffness

The CCA and CFA pressure-strain elastic modulus (EM; local stiffness) was calculated: EM = PP/(SystD − DD)/DD; cPP and pPP were considered to quantify CCA EM and CFA EM, respectively. Aortic regional stiffness was assessed by means of carotid–femoral pulse wave velocity (cfPWV) (SphygmoCor-CvMS) [4]. Real cfPWV was obtained by multiplying measured cfPWV by 0.8. cfPWV values were obtained as the median of three measurements.

### 2.8. Data Analysis

A step-wise analysis was performed. First, variables were standardized and expressed as z-scores. To this end, subjects not exposed to CRFs were selected (reference subgroup: 400 children) (Supplementary Table S1). Working with the reference subgroup, the mean value (MV) and standard deviation (SD) were determined for each arterial and hemodynamic variable (considering age and sex). Then, individual data were converted into z-scores (dimensionless numbers obtained by subtracting the reference MV from the observed value and dividing the result by the reference SD). Second, Pearson product-moment correlations were obtained to quantify the strength of association between CV z-scores and anthropometric indexes (Δz-BW, Δz-BH, Δz-BWH, Δz-BMI) considering the defined time intervals (growth periods). Third, statistical comparisons of the correlations’ strengths were done using a two-tailed William’s test, making statistical corrections for dependent (same cohort) and overlapping (correlations with a variable in common) variables (e.g., when comparing R for Δz-BW 0–6 m and z-pSBP with that for Δz-BH0–6 m and z-pSBP).

Fourth, multiple linear regression (MLR; input: forward) models enabled us to analyze the association between arterial z-scores (dependent variables) and (1) single, specific anthropometric changes, (2) z-BWH at birth; (3) current z-BMI, (4) CRFs and (5) the interactions between growth-related changes and other factors (e.g., Δz-BW 0–24 m* current z-BMI). In other words, since an association between postnatal growth and arterial parameters could be modified by birth or current body size, and/or by exposure to CRFs, the interaction between conditions was tested by adding product terms to the model. A variance inflation factor (VIF) <5 was selected to evaluate (discard) significant multicollinearity.

According to the central limit theorem, a normal distribution was considered (taking into account kurtosis and skewness, coefficients’ distribution and number (N) of studied subjects, with sample size ˃30) [24]. The number of subjects was largerthan the minimum N calculated considering α = 0.05 and β = 0.20. Analyses were done using MedCalc statistical software (v.18.5, MedCalc Inc., Ostend, Belgium), Cocor Statistical Package (http://comparingcorrelations.org/, accessed on 1 September 2020) and SPSS software (v.20, IBM-SPSS Inc., Chicago, IL, USA). A *p <* 0.05 was considered statistically significant.

## 3. Results

### 3.1. Subjects’Characteristics

Table 1, Table 2 and Table 3 show demographic, clinical, anthropometric and arterial data. There were no children with a family history of CV disease (0%).

### 3.2. Associations between Arterial Parameters at Age 6 Yearsand Anthropometric Changes in the First Two Yearsof Life

Table 4 and Table 5 show the results of bivariate correlations between arterial parameter z-scores and growth data, analyzed considering different anthropometric indexes and growth periods. Table 5 shows the associations for growth intervals including the third year of life. For all the intervals considered, there were positive associations (*p* ˂ 0.05) between anthropometric indexes and arterial parameters. The associations varied depending on the parameters evaluated.

Anthropometric variations within the first 6 m (0–6 m interval) showed almost no association with arterial parameter z-scores (Table 4). The associations between body changes in the first year (0–12 m) and arterial z-scores were mainly observed for Δz-BW and Δz-BWH. For both 0–6 and 0–12 m intervals, Δz-BMI was the parameter associated with z-cfPWV (Table 4). In the second year of life (12–24 m), Δz-BW was the only anthropometric index associated with arterial z-scores (Table 4).

The joint analysis of the first two years (0–24 m) showed that arterial parameters were mainly associated with z-BWH and z-BW (Table 4). The associations were observed almost exclusively for arterial structure (Table 4).

### 3.3. Associations between Arterial Parameters at Age 6 Years and Body Size Changes during the First Three Years of Life

Table 5 shows the associations between arterial and hemodynamic z-scores at 6 y and body size changes within the first 3 years of life. When the third year of life was considered, significant (*p* ˂ 0.05) associations were observed between body changes and hemodynamic data (e.g., z-pSBP, z-pDBP, z-cSBP). There were also associations between arterial and growth parameters. Δz-BW was (once again) the parameter with most association. Δz-BWH showed lower levels of association when the third year of life was considered (Table 4 and Table 5).

**Figure 2:**
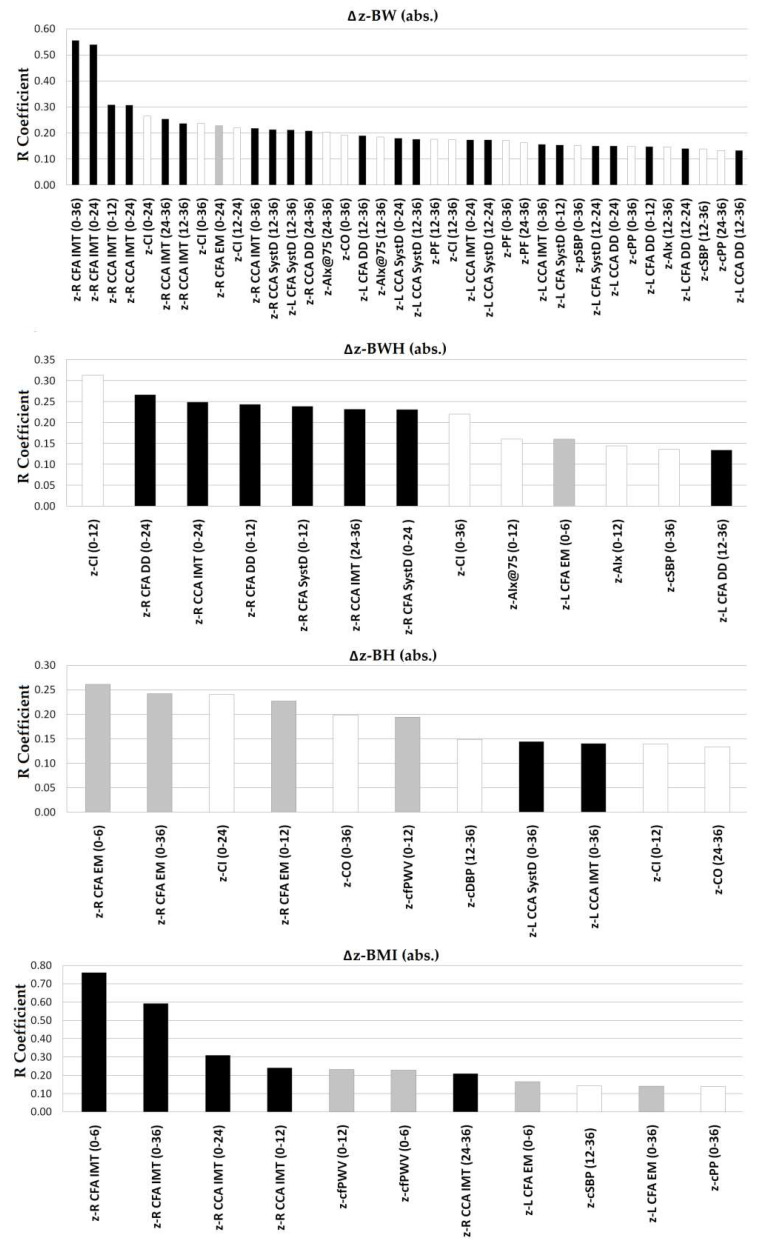
Strength of association (correlation coefficient absolute value, R) between body size changes assessed by anthropometric indexes (Δz-BW, Δz-BWH, Δz-BH, Δz-BMI) and arterial parameter z-scores at age 6 years Black bars: arterial structure parameters; white bars: hemodynamic parameters; and gray bars: arterial stiffness parameters. Numbers in brackets indicate periods in months. Only statistically significant (*p* < 0.05) correlations are shown.

Figure 2 shows, for each anthropometric index, levels (R absolute values) of the significant (*p* ˂ 0.05) associations with arterial parameters (z-scores). Changes in z-BW showed the largest number of significant associations (*n* = 38), followed by z-BWH (*n* = 13), z-BH (*n* = 11) and z-BMI (*n* = 11). Regardless of the growth interval considered, Δz-BW and Δz-BWH showed almost no association with functional arterial data. In contrast, the few associations observed for z-BH were mostly with arterial stiffness. In turn, Δz-BW, Δz-BWH and Δz-BMI were mainly associated with arterial structure (Figure 2).

### 3.4. Main Determinants of Interindividual Variations in Arterial Parameters at Age 6 Years: Role of Body Size Changes in the First Two Years of Life Considering the Exposure to CRFs

Table 6 and Table 7 show MLR results. Arterial parameter z-scores were the dependent variables, whereas anthropometric indexes, z-BWH at birth, z-BMI at age 6 years, CRFs and interactions between growth indexes and CRFs were considered independent variables. Only anthropometric indexes with statistical significance (*p <* 0.05) in bivariate tests were included (Table 4 and Table 5).

Table 6 shows MLR data, for growth intervals up to 24 m. The explanatory capacity varied depending on the time interval and arterial parameter considered. When the 0–6 m period was analyzed, HT partially explained variations in regional stiffness (z-cfPWV), whereas the interaction Δz-BMI*z-BMI contributed to explaining z-Left CFA EM.

In the first year of life (0–12 m), Δz-BW and Δz-BWH explained variations in CFA diameters. The interactions Δz-BW 0–12 m*z-BMI 6 years and Δz-BWH 0–6 m*z-pSBP contributed to explaining CFA_Left_ diameter (Table 6). However, overall, z-BMI 6 years (current z-BMI) was the variable with the major explanatory capacity for arterial z-scores at age 6 years (Table 6).

Δz-BW 0–24 m and Δz-BWH 0–24 m contributed to explaining variations in arterial structure at the age of 6 years (Table 6). When the first year of life was not considered (12–24 m interval), only z-BMI contributed to explaining variations in arterial structure at age 6 years (Table 6).

### 3.5. Main Determinants of Interindividual Variations in Arterial Parameters at Age 6 Years: Role of Body Size Changes in the First Three YearsConsidering the Exposure to CRFs

Table 7 shows MLR data for growth intervals up to 36 m. Δz-BW 0–36 m enabled us to explain (in isolation or interacting with z-pSBP) variations in peripheral and central hemodynamic parameters (i.e., z-pSBP, z-cSBP, z-cPP, z-Pf, z-CI), and arterial structure (e.g., z- CCA IMT) (Table 7).

Δz-BW 12–36 m was an explanatory variable for hemodynamic parameters like z-AIx, z-AIx@75, z-AP, z-Pf, z-CI. Additionally, Δz-BW 12–36 m contributed to explaining variations in arterial structure (Table 7). Likewise, Δz-BW 24–36 m contributed to explaining variations in z-AIx@75, z-Pf, z-CCA_Right_ DD and IMT (in isolation and interacting with z-pSBP) (Table 7).

## 4. Discussion

### 4.1. Main Findings

This work’s main findings can be summarized as follows:

First, differences in arterial parameters (z-scores) found in children at 6 years old are mainly associated with Δz-BW, when considering growth-related changes in the first two years of life (0–12, 0–24, 12–24 m) (Table 4). In contrast, variations in z-BH or z-BMI in this period showed almost no association with arterial parameters at age 6 years. Similar results were obtained for anthropometric variations in the growth period from 0 to 6 m.

Second, Δz-BW and Δz-BWH in the first two years of life were associated with arterial structure variations at age 6 y, but did not explain variations in hemodynamic parameters (i.e., pBP or cBP) (Table 4). The associations were independent of CRFs, z-BWH at birth and current z-BMI (Table 6).

Third, when the third year of life was considered, Δz-BW was associated with arterial parameters (Table 4 and Table 5). The associations were independent of current z-BMI (Table 7).

Fourth, Δz-BW (for different growth intervals) was the anthropometric index with a major association with arterial parameters (z-scores) at age 6 years (Table 6 and Table 7). z-BW changes after the first year showed the greatest associations with arterial parameter z-scores at age 6 years.

Anthropometry is a widely used, inexpensive and non-invasive measure of the nutritional status of an individual or population. It serves different purposes, related tothe anthropometric indicators selected. Data usually considered for anthropometric assessment are: age, sex, BW and BH (or length, when ˂2 years). The different variables provide complimentary information. When used together, they can provide considerable data about a person’s nutritional status and, when two variables are used together, they define an “anthropometric index” [25]. Indexes commonly used to assess the nutritional status of children and adolescents are: (1) BW for age; (2) BH (or length) for age, (3) BW for BH (BWH) and/or (4) BMI. The indexes described are used to measure nutritional imbalance (e.g., undernutrition, stunting, overweight/obesity). Advantages and disadvantages have been ascribed to each of the different indexes. BW for age, an index widely recommended to evaluate underweight, has the advantage of being a parameter that reflects both past (chronic) and present (acute) undernutrition, but it is unable to differentiate them [25]. BHforage is used to identify past or chronic malnutrition (i.e., stunting), but it cannot indicate short-term alterations [25]. BW for BH helps to identify children with current or acute undernutrition or wasting and is useful for screening at risk children, and for measuring short-term changes in nutritional status. In this regard, it is appropriate when examining short-term effects (e.g., seasonal changes in food supply, nutritional stress brought about by illness) [25]. Finally, BMI (or Quetelet’s index), is considered an index of body fat and protein stores [25]. Children and adolescents or adults with a healthy nutritional status would be expected to have body stores (z-BMI or BMI) within a certain range. Understanding the meaning and use of the different anthropometric indicators would help to define and select the most appropriate for an evaluation, depending on its aims. Current WHO recommendations suggest using z-BWH to followup children in the first 2 years of life, whereas beyond 5 y, the recommended index is z-BMI [26]. However, it is unknown which index and growth interval would be the most useful to explain and identify arterial interindividual variations in children.

In this work, we found that variations in z-BW were the anthropometric data with major associations with arterial parameters assessed at age 6 years. The associations were mainly with arterial structure in the first two years of life, and with arterial structure, stiffness and hemodynamics when the third year of life was included in the analysis.

The 38 significant associations observed for Δz-BW (Table 4 and Table 5) were distributed as follows among the growth intervals analyzed: 0–6 m (*n* = 0), 0–12 m (*n* = 3), 0–24 m (*n* = 7), 0–36 m (*n* = 8), 12–24 m (*n* = 4), 12–36 m (*n* = 11) and 24–36 m (*n* = 5). Therefore, most of the associations were obtained for body changes in periods of at least 2 y (0–24, 0–36, 12–36 m.). Only a few (*n* = 3) associations were observed for body changes in the first year of life. Then, it could be said that changes in z-BW involving more than a year and including the second and third year of life would show the greatest association with arterial properties (z-scores) at age 6 years. In agreement with other studies, the long-term impact of accelerated growth in infancy and early childhood would depend on its timing and duration. Subjects with high rates of body size growth show high levels of exposure to CRFs (e.g., obesity [9,10,11,12]; high pBP [13], adverse lipid profile [9,11], impaired insulin sensitivity [9,11]), making it difficult to determine whether (potential) arterial variations are directly or indirectly associated with growth rates. In addition, there is a lack of data regarding the impact of postnatal growth patterns on arterial parameters in early childhood.

In this work, the associations between anthropometric indexes and hemodynamic and arterial parameter z-scores were independent or interacted with exposure to CRFs and body characteristics at birth and/or at the time of the study. Early childhood is a lifeperiod in which the cumulative impact of factors related to increased CV risk (e.g., HT) would be small. Further studies would be necessary to analyze whether the association between anthropometric variations and arterial parameter z-scores is maintained as the age increases. Recently, we demonstrated that the association between growth-related anthropometric changes and arterial z-scores was greater when the arterial system was evaluated at age 6 years than at age 18–19 years [4]. Associations between early body changes and CV parameters would gradually decrease with increasing age, andthe exposure to CRFs (e.g., smoke, HT) would probably show a greater capacity to explain arterial parameters [4].

In our work, BW changes in the first years of life were associated mainly with arterial structure (rather than with arterial stiffness or hemodynamics). Our findings partially agree with Evelein et al. [5] who showed that in healthy children (5 y), a history of a large (in excess) BW and BWH gain in the early postnatal period (0–3 m) was associated with increased carotid IMT, but not with arterial stiffness. The association was not modified by BW at birth, or explained by pBP levels. When the analyses were done considering later growth periods (3–6, 6–9 and 9–12 m) the authors did not find associations with the arterial parameters. The authors analyzed short time periods (3 m in length), which, taking into account our findings, could have limited the ability to find associations. Linhares et al. reported that relative BW gain within 2–4 years was associated with higher IMT [27]. The authors did not find an association between BW gain or linear growth (BH gain) within the first 2 y (0–24 m) and carotid IMT. This is in agreement with our findings. Skilton et al. reported that BW gain in the period from 0 to 18 m was positively associated with carotid extra-medial thickness in subjects aged 8 y [14]. Toemen et al. [28] studied children with different intrauterine growth patterns and found higher pSBP levels in those who had normal intrauterine growth and accelerated growth in the first years of life. Therefore, the association between growth-related body changes and hemodynamic parameters could not depend on initial conditions and could be observed in healthy children. This is in agreement with our findings.

### 4.2. Clinical Implications

Recently, there has been increased awareness of the importance of nutrition for human health and wellbeing. Key indicators of a population’s nutritional status are based on anthropometric data. Therefore, the selection and use of accurate anthropometric data are critical to provide reliable information (e.g., to policy makers, program executives, researchers) [29]. The quality of anthropometric data is also important in analyzing the implementation and impact of health and nutritional interventions [29]. In this context, our work suggests that the most appropriate variable, in terms of association with the arterial status at age 6 years, would be the variations in z-BW (followed by changes in z-BWH), assessed considering time intervals of more than a year and including the second and third year of life.

Although the effect size (R value) of infant growth on arterial parameter interindividual variations would be small and (possibly) without clinical meaning for the individuals, the findings would be important at the population level. This work’s results would contribute to the knowledge of developmental origins of arterial disease. They suggest that rates of body size growth within the first two and three years of life could impact on arterial parameters that could be related (at least in theory) to increased CV risk, even more so if the phenomenon of carrying conditions and/or patterns is maintained (or amplified) as age increases. This subject requires further study [4,28].

### 4.3. Strengths and Limitations

This work is a population-based prospective cohort design, which included a large number of subjects, studied from early life stages. Repeated measures during the growth period enabled us to study the association of variations in growth profiles and arterial parameters. Our own specific “reference population” was considered to define arterial parameters z-scores (Supplementary Table S1) [4]. Potential anthropometric and non-anthropometric confounders (cofactors) were considered to isolate the effect of bodysize change in the statistical models [30]. Taking into account that the impact of body size change on the arterial system could depend on the time interval in which it occurs, we analyzed different time intervals. The relationship between body size changes and pBP has been one of the most studied (based on the “fetal origin” hypothesis). However, pBP is a single parameter and does not inform us about central hemodynamic conditions [17], arterial structure and/or stiffness (e.g., associated with early vascular aging or atherosclerosis development). Thus, we designed an integral approach in which different hemodynamic variables (central and peripheral), arteries (e.g., elastic (carotid) and muscular (femoral)) and arterial parameters were evaluated. Unlike most works that analyzed the associations between body size changes and the CV system considering premature, small for gestational age, obese and/or hypertensive subjects, in this work, we studied healthy children.

Some limitations should be considered. First, we did not have blood biomarker measurements made by our technicians. Therefore, data about some conditions (e.g., dyslipidemia) wereobtained from reference physicians, registers and/or self-reports. Second, although we adjusted for several potential confounders, residual confounding factors may persist, as in any observational study. Third, in this work, we opted for using changes in z-BW, z-BH, z-BWH or z-BMI between two time points as growthindicators. This approach is a simple practical (clinical) method to quantify a “change”, although more detailed growth patterns could be derived from longitudinally collected anthropometric data. Being a cross-sectional study, this work did not allow for establishing causal relationships between anthropometric changes and/or CRFs and the arterial parameters evaluated at age 6 years. Fourth, we did not do an analysis discriminating by sex, althoughwe are aware of data suggesting that the impact of childhood growth on the CV system may differ between boys and girls [8]. Fifth, we included subjects born at term and preterm, but as most of them belonged to the first condition, results should be assigned to term-born subjects. Finally, making several correlations (e.g., Table 4) increases the risk of type I errors. Aiming at minimizing this, the level of statistical significance of the correlation coefficients could be adjusted (e.g., Bonferroni correction). However, correction can lead to type II errors. After analyzing the pros and cons of making adjustments and considering the research context, we decided not to make them, mainly due to the following reasons. First, it would increase the risk of type II errors. As an example, in Table 5, there are 33 correlations for each CV variable and, therefore, a threshold *p* value (corrected) equal to 0.0015 (0.05/33 = 0.0015) should be considered. Most of the correlations showed very low *p* values (e.g., *p* ˂ 0.001), which clearly have biological theoretical explanations. Second, our results represent exploratory research/data analysis, rather than confirmatory analysis. They were not presented with the aim of stimulating final decision making. Future works should be developed before reaching definitive conclusions. Third, correlation coefficients are effect sizes, so in real terms, we do not need a *p* value to interpret them. Finally, for any age, arterial properties can be acutely and temporarily modified by variations in the vascular smooth muscle (VSM) tone [31,32,33,34,35]. Systematization of recording conditions is necessary for the evaluation of arterial parameters considering the modulating role of the VSM tone. In this work, to systematize the records and as a way to minimize the impact of the known source of variability, arterial parameters were assessed and determined at rest, under stable hemodynamic conditions.

## 5. Conclusions

First, when considering growth-related anthropometric variations in the first two years of life, variations in arterial parameters (z-scores) in 6-year-old children were mainly associated with Δz-BW. Variations in z-BH or z-BMI in that period showed almost no association with arterial parameters. Similarly, anthropometric variations in the 0–6 m period showed few associations with arterial parameters. Second, Δz-BW and Δz-BWH in the first two years of life were associated with arterial structure at the age 6 y, but not with pBP or cBP. The associations were independent of cofactors (e.g., CRFs, z-BWH at birth and current z-BMI). Third, when the third year of life was considered, Δz-BW was the anthropometric variable mainly associated with arterial parameters at age 6 years.

Changes in z-BW involving periods of more than a year and including the second and third year of life showed the greatest associations with arterial parameters in early childhood.

## Figures and Tables

**Table 1:** Children´s characteristics at age 6 years (*n* = 632; female: 49.5%).

	MV	SD	Min.	p25th	p50th	p75th	Max.
Age (years)	6.02	0.3	5.07	5.80	6.04	6.26	6.66
BW at birth (Kg)	3.27	1.45	0.98	2.95	3.28	3.59	5.00
BW (kg)	22.31	4.67	14.00	19.20	21.25	24.35	46.50
BH (m)	1.14	0.05	0.99	1.10	1.14	1.17	1.33
BMI (Kg/m^2^)	17.03	2.48	12.10	15.37	16.43	18.06	27.15
z-BMI (current or “~6 years”) (SD)	0.96	1.47	−2.60	0.06	0.66	1.62	7.37
z-BW (current or “~6 years”) (SD)	0.45	1.30	−2.39	−0.45	0.32	1.18	6.15
z-BH (current or “~6 years”) (SD)	−0.33	1.04	−3.43	−1.05	−0.34	0.29	3.55
Obesity (*n*, %)	108 (17.1)						
Dyslipidemia (*n*, %)	1 (0.2)						
Diabetes (*n*, %)	1 (0.2)						
Hypertension (*n*, %)	27 (4.3)						
Mode of delivery (cesarean; *n*, %)	202 (31.9)						
Preterm birth (*n*, %)	62 (9.8)						
Mother, diabetes (*n*, %)	12 (1.9)						

MV: mean value. SD: standard deviation. Min., Max.: minimal and maximal value. z: z-score. p25th, p50th, p75th: 25th, 50th and 75^th^ percentile. BW and BH: body weight and height. BMI: body mass index.

**Table 2:** Body size during growth (*n* = 632).

	MV	SD	Min.	p25th	p50th	p75th	Max.
z-BWH at birth (SD)	0.40	1.14	−4.06	−0.27	0.49	1.13	3.61
z-BW at birth (SD)	0.02	0.95	−5.24	−0.56	0.06	0.68	2.95
z-BH at birth (SD)	−0.27	1.13	−4.91	−1.00	−0.08	0.46	4.75
z-BMI at birth (SD)	0.27	1.06	−3.59	−0.38	0.36	0.93	3.65
z-BWH 6 m.(SD)	0.59	1.26	−3.86	−0.20	0.50	1.29	4.76
z-BW 6 m.(SD)	0.38	1.14	−2.83	−0.44	0.32	1.08	4.54
z-BH 6 m.(SD)	−0.15	1.11	−4.12	−0.92	−0.19	0.60	3.30
z-BMI 6 m. (SD)	0.66	1.29	−4.07	−0.15	0.58	1.40	5.42
z-BWH 12 m. (SD)	0.33	1.08	−2.78	−0.40	0.32	0.92	3.91
z-BW 12 m. (SD)	0.64	1.16	−2.77	−0.14	0.58	1.37	5.51
z-BH 12 m. (SD)	−0.31	1.22	−4.72	−1.06	−0.39	0.38	5.57
z-BMI 12 m. (SD)	0.69	1.19	−2.79	−0.09	0.63	1.44	5.84
z-BWH 24 m. (SD)	0.59	1.26	−3.86	−0.20	0.50	1.29	4.76
z-BW 24 m. (SD)	0.38	1.14	−2.83	−0.44	0.32	1.08	4.54
z-BH 24 m. (SD)	−0.15	1.11	−4.12	−0.92	−0.19	0.60	3.30
z-BMI 24 m. (SD)	0.66	1.29	−4.07	−0.15	0.58	1.40	5.42
z-BWH 36 m. (SD)	0.59	1.25	−3.45	−0.22	0.47	1.37	6.43
z-BW 36 m. (SD)	0.35	1.14	−2.39	−0.46	0.29	1.07	5.00
z-BH 36 m. (SD)	−0.11	1.14	−3.92	−0.89	−0.16	0.56	3.30
z-BMI 36 m. (SD)	0.60	1.28	−3.87	−0.21	0.48	1.39	6.48

Abbreviations: identical to Table 1.

**Table 3:** Arterial parameters at age 6 years (*n* = 632).

	MV	SD	Min.	p25th	p50th	p75th	Max.
Central and peripheral hemodynamic parameters							
Heart rate (beats/minute)	91	11	66	84	90	99	134
pSBP (mmHg)	100	8	80	94	100	105	126
pDBP (mmHg)	59	7	50	54	58	62	86
pPP (mmHg)	41	7	24	36	40	46	77
pMBP (mmHg)	72	7	31	68	73	77	96
cSBP (mmHg)	83	6	64	78	83	87	100
cDBP (mmHg)	60	6	46	56	60	64	77
cPP (mmHg)	22	4	7	19	22	25	43
CO (liter/minute)	4.4	0.3	3.2	4.1	4.4	4.8	5.7
CI (liter/minute/m^2^)	4.9	0.6	3.5	4.4	4.9	5.4	5.9
SVR (s.mmHg/mL)	1.10	0.10	0.86	1.04	1.11	1.19	1.41
AIx (%)	9.7	9.7	−16	3	10	17	37
AIx@75 (%)	17.0	9.3	−10	11	17	23	43
AP (mmHg)	2	2	−5	1	2	4	9
Pf (mmHg)	20	5	7	17	19	23	43
Pb (mmHg)	10	4	3	9	10	11	78
Structural arterial parameters							
CCA_Right_ SystD (mm)	6.04	0.50	4.83	5.70	5.98	6.39	7.41
CCA_Right_ DD (mm)	5.38	0.47	4.21	5.03	5.36	5.71	6.91
CCA_Right_ IMT (mm)	0.421	0.028	0.370	0.405	0.418	0.431	0.537
CCA_Left_ SystD (mm)	5.92	0.46	4.84	5.59	5.91	6.22	7.48
CCA_Left_ DD (mm)	5.25	0.43	4.25	4.96	5.24	5.53	6.99
CCA_Left_ IMT (mm)	0.420	0.027	0.316	0.405	0.417	0.431	0.567
CFA_Right_ SystD (mm)	4.74	0.53	3.44	4.42	4.75	5.05	6.50
CFA_Right_ DD (mm)	4.42	0.51	3.13	4.11	4.43	4.72	6.11
CFA_Right_ IMT (mm)	0.331	0.032	0.282	0.313	0.323	0.346	0.411
CFA _Left_ SystD (mm)	4.72	0.50	3.51	4.36	4.70	5.01	6.37
CFA_Left_ DD (mm)	4.40	0.50	3.29	4.06	4.38	4.71	5.98
CFA_Left_ IMT (mm)	0.335	0.026	0.271	0.319	0.331	0.355	0.398
Local and regional arterial stiffness							
CCA_Right_ EM (mmHg)	187	45	71	156	188	216	304
CCA_Left_ EM (mmHg)	179	46	54	146	175	205	314
CFA_Right_ EM (mmHg)	618	267	237	417	564	724	1555
CFA_Left_ EM (mmHg)	592	219	219	427	546	711	1561
cfPWV (m/s)	4.81	0.76	2.88	4.28	4.75	5.25	7.72

MV: mean value. SD: standard deviation. Min., Max.: minimal, maximal, respectively. p25th, p50th, p75th: 25th, 50th, 75th percentile, respectively. SBP, DBP, PP, MBP: systolic, diastolic, pulse and mean pressure, respectively (p: peripheral, c: central). SVR: systemic vascular resistance. CO, CI: cardiac output and index, respectively. AIx, AIx@75: augmentation index without and with heart rate correction (75 beats/minute), respectively. AP: augmented pressure. Pf, Pb: forward and backward pressure wave components, respectively. DD, SystD: diastolic and systolic diameter. CCA, CFA: common carotid and common femoral artery, respectively. EM: elastic modulus. IMT: intima–media thickness. cfPWV: carotid–femoral pulse wave velocity.

**Table 4:** Comparison of correlations between arterial z-scores at age 6 years and anthropometric variations in different growth periods (0–6, 0–12, 0–24 and 12–24 m).

	Zero-Order Correlations									William’s Test (Correlation Comparisons)					
0–6 m	ΔzBWH [1]		ΔzBW [2]			ΔzBH [3]		ΔzBMI [4]		1 vs. 2	1 vs. 3	1 vs. 4	2 vs. 3	2 vs. 4	3 vs. 4
	R	p	R	p		R	p	R	p	p	P	*p*	P	*p*	P
Structural arterial parameters															
z-CFA_R_ IMT	0.56	0.17	0.18	0.113		0.10	0.715	0.76	0.0004	0.117	0.101	<0.0001	0.717	<0.0001	0.011
Local and regional arterial stiffness															
z-CFA_R_ EM	−0.12	0.27	0.14	0.213		0.26	0.019	−0.05	0.666	0.001	0.012	0.012	0.208	0.005	0.040
z-CFA_L_ EM	−0.16	0.024	−0.11	0.139		0.04	0.556	−0.16	0.022	0.310	0.041	0.990	0.017	0.251	0.041
z-cfPWV	−0.16	0.077	−0.03	0.726		0.19	0.320	−0.23	0.011	0.040	0.003	0.002	0.006	0.000	0.007
0–12 m	ΔzBWH [1]		ΔzBW [2]			ΔzBH [3]		ΔzBMI [4]		1 vs. 2	1 vs. 3	1 vs. 4	2 vs. 3	2 vs. 4	3 vs. 4
	R	p	R	p		R	p	R	p	*p*	p	*p*	P	p	P
Central and peripheral hemodynamic parameters															
z-AIx	−0.14	0.031	−0.02	0.800		−0.08	0.247	0.01	0.871	0.002	0.419	<0.0001	0.397	0.286	0.230
z-AIx@75	−0.16	0.019	−0.01	0.841		−0.09	0.204	−0.01	0.921	0.002	0.359	<0.0001	0.274	0.783	0.286
z-CI	−0.31	0.000	−0.04	0.611		−0.14	0.047	−0.09	0.203	<0.0001	0.021	<0.0001	0.169	0.089	0.522
Structural arterial parameters															
z- CCA_R_ IMT	0.14	0.180	0.31	0.002		0.18	0.090	0.24	0.021	0.033	0.730	0.094	0.221	0.099	0.600
z-CFA_R_ SystD	0.24	0.024	0.18	0.088		0.12	0.268	0.17	0.133	0.458	0.317	0.240	0.586	0.827	0.681
z- CFA_R_ DD	0.24	0.022	0.19	0.075		0.16	0.150	0.16	0.157	0.536	0.502	0.198	0.784	0.511	0.983
z-LCFA_L_SystD	0.09	0.207	−0.15	0.030		0.04	0.600	−0.11	0.138	<0.0001	0.525	<0.0001	0.010	0.172	0.057
z-CFA_L_ DD	0.08	0.232	−0.15	0.038		0.03	0.688	−0.10	0.161	<0.0001	0.526	<0.0001	0.009	0.088	0.099
Local and regional arterial stiffness															
z-CFA_R_ EM	0.05	0.618	0.12	0.253		0.23	0.038	0.04	0.738	0.204	0.021	0.807	0.124	0.498	0.015
z-cfPWV	−0.02	0.840	−0.11	0.236		0.19	0.030	−0.23	0.009	0.208	0.037	0.000	0.002	<0.0001	<0.0001
0–24 m	Δz-BWH [1]		Δ z-BW [2]			Δ z-BH [3]		Δ z-BMI [4]		1 vs. 2	1 vs. 3	1 vs. 4	2 vs. 3	2 vs. 4	3 vs. 4
	R	p	R	p		R	p	R	p	*p*	p	p	P	*p*	P
Central and peripheral hemodynamic parameters															
z-CI	−0.08	0.263	−0.26	0.0001		−0.24	0.001	−0.08	0.279	<0.0001	0.071	0.990	0.696	<0.0001	0.069
Structural arterial parameters															
z-CCA_R_ IMT	0.25	0.016	0.31	0.004		0.20	0.069	0.31	0.004	0.696	0.870	0.344	0.542	0.997	0.718
z-CCA_L_ SystD	0.03	0.702	0.18	0.011		0.09	0.204	0.11	0.118	0.006	0.499	<0.0001	0.078	0.076	0.821
z-CCA_L_ DD	−0.01	0.940	0.15	0.034		0.06	0.432	0.12	0.108	0.000	0.432	<0.0001	0.080	0.448	0.498
z-CCA_L_ IMT	0.06	0.421	0.17	0.014		0.13	0.061	0.10	0.174	0.012	0.430	0.029	0.434	0.078	0.734
z-CFA_R_ SystD	0.23	0.033	0.12	0.288		0.07	0.524	0.09	0.435	0.112	0.248	<0.0001	0.544	0.637	0.888
z-CFA_R_ DD	0.27	0.013	0.16	0.156		0.11	0.362	0.12	0.304	0.109	0.240	<0.0001	0.542	0.527	0.943
z-CFA_R_ IMT	−0.12	0.635	0.54	0.038		0.40	0.152	0.17	0.558	<0.0001	0.092	<0.0001	0.381	<0.0001	0.439
Local and regional arterial stiffness															
z-CFA_R_ EM	0.15	0.160	0.23	0.044		0.19	0.096	0.14	0.228	0.072	0.651	0.591	0.440	0.025	0.577
12–24 m	Δz-BWH [1]		Δ z-BW [2]		Δ z-BH [3]			Δ z-BMI [4]		1 vs. 2	1 vs. 3	1 vs. 4	2 vs. 3	2 vs. 4	3 vs. 4
	R	p	R	p	R		p	R	p	*p*	p	p	P	*p*	P
Central and peripheral hemodynamic parameters															
z-Cardiac Index	0.08	0.264	−0.22	0.001	−0.07		0.321	−0.04	0.557	<0.0001	0.069	0.081	0.162	<0.0001	0.685
Structural arterial parameters															
z-CCA_L_ SystD	−0.04	0.576	0.17	0.010	−0.01		0.879	0.09	0.212	0.032	0.717	0.208	0.204	0.183	0.360
z-CCA_L_ DD	−0.04	0.553	0.12	0.083	−0.01		0.857	0.05	0.425	0.104	0.717	0.385	0.362	0.247	0.589
z-CFA_L_ SystD	−0.07	0.325	0.15	0.029	−0.09		0.175	0.06	0.360	0.001	0.808	0.061	0.012	0.026	0.044
z-CFA_L_ DD	−0.08	0.220	0.14	0.043	−0.08		0.221	0.04	0.523	0.001	0.990	0.083	0.021	0.013	0.107
z-CFA_L_ IMT	0.16	0.286	0.11	0.481	0.08		0.608	0.12	0.430	0.445	0.330	0.559	0.021	0.805	0.589

z: z-score. BW, BH, BWH: body weight, body height and BW for BH, respectively. BMI: body mass index Δ: change in the analyzed period. SBP, DBP, PP and MBP: systolic, diastolic, pulse and mean blood pressure, respectively (p: peripheral, c: central). CI: cardiac index. SVR: systemic vascular resistance. AIx, AIx@75: aortic augmentation index without and with heart rate correction (75 beats/minute). AP: augmented pressure. Pf, Pb: forward and backward aortic component. DD, SystD: diastolic and systolic diameter. CCA, CFA: common carotid and femoral artery. EM: elastic modulus. IMT: intima–media thickness (suffix R and L: right and left). cfPWV: carotid–femoral pulse wave velocity. R: Pearson coefficient. A *p <* 0.05 was considered significant. Statistical analysis: zero-order correlations and William’s coefficient for correlation comparison. Only the correlations with statistical significance are shown.

**Table 5:** Comparison of correlations between arterial z-scores at age 6 years and anthropometric variations in different growth periods (0–36, 12–36, 24–36 m).

	Zero-Order Correlations								William’s Correlation Comparisons					
0–36 m	Δz-BWH [1]		Δz-BW [2]		Δz-BH [3]		Δz-BMI [4]		1 vs. 2	1 vs. 3	1 vs. 4	2 vs. 3	2 vs. 4	3 vs. 4
	R	p	R	p	R	p	R	P	p	p	P	P	p	P
Central and peripheral hemodynamic parameters														
z-pSBP	0.09	0.177	0.15	0.032	0.13	0.076	0.08	0.253	0.324	0.660	0.893	0.713	0.002	0.499
z-cSBP	0.14	0.041	0.13	0.051	0.08	0.268	0.10	0.154	0.838	0.486	0.567	0.334	0.463	0.776
z-cPP	0.10	0.127	0.15	0.028	0.05	0.433	0.14	0.040	0.277	0.564	0.567	0.067	0.816	0.222
z-Pf	0.07	0.296	0.17	0.012	0.13	0.054	0.07	0.336	0.029	0.487	0.990	0.441	0.015	0.396
z-Cardiac Output	0.07	0.319	0.19	0.005	0.20	0.004	0.06	0.372	0.009	0.127	0.892	0.901	0.002	0.054
z-Cardiac Index	−0.22	0.001	−0.24	0.0004	−0.10	0.144	−0.13	0.060	0.671	0.176	0.212	0.009	0.009	0.683
Structural arterial parameters														
z- CCA_Right_ IMT	0.12	0.256	0.22	0.035	0.20	0.059	0.17	0.113	<0.0001	0.368	0.648	0.802	0.431	0.782
z-CCA_Left_ SystD	0.01	0.830	0.13	0.070	0.14	0.039	−0.03	0.641	0.099	0.342	0.720	0.891	0.013	0.125
z- CCA_Left_ IMT	0.11	0.125	0.16	0.024	0.14	0.045	0.05	0.447	0.490	0.825	0.588	0.811	0.087	0.415
z-CFA_Right_ IMT	0.21	0.382	0.55	0.017	0.16	0.536	0.59	0.009	0.023	0.705	0.041	0.021	0.744	0.054
Local and regional arterial stiffness														
z- CFA_Right_ EM	−0.02	0.850	0.18	0.092	0.24	0.026	−0.01	0.897	<0.0001	0.004	0.893	0.262	<0.0001	0.029
z- CFA_Left_ EM	−0.08	0.263	−0.06	0.436	0.07	0.302	−0.14	0.047	0.793	0.296	0.730	0.128	0.064	0.069
12–36 m	Δz-BWH [1]		Δz-BW [2]		Δz-BH [3]		Δz-BMI [4]		1 vs. 2	1 vs. 3	1 vs. 4	2 vs. 3	2 vs. 4	3 vs. 4
Central and peripheral hemodynamic parameters														
z-cSBP	0.05	0.485	0.14	0.033	−0.09	0.159	0.14	0.029	0.154	0.120	0.133	0.002	0.940	0.199
z-cDBP	−0.04	0.528	0.05	0.471	−0.15	0.023	0.06	0.393	0.156	0.033	0.097	0.002	0.804	<0.0001
z-AIx	0.03	0.698	−0.15	0.025	0.06	0.361	−0.09	0.193	0.044	0.725	0.035	0.008	0.114	<0.0001
z-AIx@75	0.01	0.893	−0.18	0.006	0.02	0.800	−0.10	0.125	0.014	0.907	0.053	0.008	0.034	0.419
z-AP	0.03	0.604	−0.11	0.082	0.06	0.355	−0.06	0.350	0.019	0.725	0.115	0.004	0.189	0.001
z-PF	0.04	0.596	0.18	0.007	0.05	0.418	0.10	0.122	0.024	0.903	0.297	0.031	0.034	0.181
z-Cardiac Index	0.08	0.251	−0.17	0.008	0.02	0.743	−0.06	0.381	0.001	0.502	0.019	0.001	0.006	0.040
Structural arterial parameters														
z- CCA_Right_ SystD	0.14	0.180	0.21	0.034	0.02	0.834	0.19	0.060	0.020	0.369	0.572	0.002	0.735	0.003
z- CCA_Right_ DD	0.16	0.112	0.20	0.050	0.03	0.737	0.19	0.057	0.662	0.329	0.734	0.070	0.866	0.005
z- CCA_Right_ IMT	−0.09	0.354	−0.24	0.018	−0.02	0.841	−0.15	0.128	0.106	0.602	0.501	0.018	0.089	0.025
z- CCA_Left_ SystD	−0.03	0.700	0.18	0.008	0.02	0.817	0.08	0.226	0.001	0.569	0.061	0.009	0.010	0.116
z- CCA_Left_ DD	−0.01	0.829	0.13	0.047	0.01	0.826	0.06	0.342	0.023	0.820	0.233	0.009	0.074	0.190
z- CFA_Left_ SystD	−0.12	0.075	0.21	0.002	0.01	0.866	0.03	0.679	<0.0001	0.137	0.013	0.001	<0.0001	0.601
z- CFA_Left_ DD	−0.13	0.049	0.19	0.005	0.01	0.848	0.01	0.898	<0.0001	0.109	0.020	0.003	<0.0001	0.915
z- CFA_Left_ IMT	0.17	0.241	0.16	0.272	0.14	0.357	0.13	0.376	0.873	0.726	0.501	0.749	0.453	0.797
24–36 m	Δz-BWH [1]		Δz-BW [2]		Δz-BH [3]		Δz-BMI [4]		1 vs. 2	1 vs. 3	1 vs. 4	2 vs. 3	2 vs. 4	3 vs. 4
	R	P	R	p	R	p	R	P	p	p	P	P	p	p
Central and peripheral hemodynamic parameters														
z-cPP	0.09	0.191	0.13	0.043	0.07	0.294	0.07	0.304	0.319	0.836	0.739	0.402	0.178	0.992
z-AIx@75	−0.09	0.186	−0.20	0.002	−0.11	0.107	−0.09	0.194	0.004	0.835	0.990	0.192	0.011	0.836
z-PF	0.09	0.177	0.16	0.014	0.08	0.260	0.08	0.224	0.067	0.918	0.954	0.241	0.060	0.950
z-Cardiac Output	0.02	0.821	0.06	0.349	0.13	0.049	0.00	0.979	0.525	0.264	0.765	0.324	0.193	0.236
Structural arterial parameters														
z- CCA_Right_ DD	0.13	0.192	0.21	0.044	0.06	0.594	0.13	0.229	0.196	0.651	0.990	0.175	0.244	0.651
z- CCA_Right_ IMT	−0.23	0.024	−0.25	0.014	0.01	0.946	−0.21	0.045	0.743	0.116	0.826	0.018	0.555	0.153

z: z-score. BW, BH, BWH: body weight, body height, BW for BH, respectively. BMI: body mass index Δ: change in the analyzed period. SBP, DBP, PP and MBP: systolic, diastolic, pulse and mean blood pressure, respectively (p: peripheral, c: central). SVR: systemic vascular resistance. AIx, AIx@75: aortic augmentation index without and with heart rate correction (75 beats/minute). AP: augmented pressure. Pf, Pb: forward and backward wave component, respectively. DD, SystD: diastolic and systolic diameter, respectively. CCA, CFA: common carotid and common femoral artery, respectively. EM: elastic modulus. IMT: intima–media thickness. cfPWV: carotid–femoral pulse wave velocity. R: Pearson coefficient. A *p <* 0.05 was considered significant. Statistical analysis: zero-order correlations and William’s coefficient for correlation comparison. Only the correlations in which the CV variable reached statistical significance with anthropometric variables are shown.

**Table 6:** Multiple linear regression (MLR) analysis between arterial parameter z-scores at age 6 years (dependent variables) and anthropometric indexes and cardiovascular risk factors (CRFs) (independent variables).

Dependent Variable	Independent Variables	βu	SE	βs	P	Adj R^2^
Δz-BMI (0–6 m) included as an independent variable						
z-CFA_Left_ EM	Constant	0.088	0.081		0.278	0.031
	Δz-BMI 0–6 m* z-BMI	−0.072	0.031	−0.194	0.023	
z-cfPWV	Constant	−0.076	0.100		0.448	0.041
	Hypertension	1.333	0.542	0.223	0.015	
Δz-BW (0–12 m) included as an independent variable						
z-CCA_Right_ IMT	Constant	−0.830	Lo		0.496	0.131
	Δz-BW 0–12 m	0.317	0.085	0.376	<0.001	
z-CFA_Right_ SystD	Constant	−0.126	0.149		0.401	0.142
	z-BMI	0.395	0.110	0.384	0.010	
	z-BWH at birth	−0.241	0.111	−0.233	0.033	
z-CFA_Right_ DD	Constant	−0.114	0.148		0.443	0.135
	z-BMI	0.376	0.110	0.369	0.001	
	z-BWH at birth	−0.248	0.111	−0.241	0.028	
z-CFA_Left_ SystD	Constant	−0.174	0.112		0.121	0.055
	z-BMI	0.236	0.079	0.267	0.030	
	Δz-BW 0–12 m * z-BMI	−0.077	0.360	−0.187	0.038	
z-CFA_Left_ DD	Constant	−0.145	0.110		0.190	0.051
	z-BMI	0.220	0.078	0.254	0.005	
	Δz-BW 0–12 m* z-BMI	−0.078	0.036	−0.193	0.032	
Δz-BWH (0–12 m) included as an independent variable						
z-AIx	Constant	−0.990	0.068		0.149	0.140
	z-pSBP	−0.289	0.059	−0.320	<0.001	
	Δz-BWH 0–12 m	−0.133	0.045	−0.193	0.004	
z-AIx@75	Constant	−0.062	0.069		0.366	0.109
	z-pSBP	−0.257	0.061	−0.289	<0.001	
	Δz-BWH 0–12 m	−0.113	0.047	−0.166	0.017	
z-CFA_Left_ SystD	Constant	−0.142	0.108		0.192	0.067
	z-BMI	0.193	0.070	0.219	0.007	
	Δz-BWH 0–12 m* z-pSBP	−0.118	0.048	−0.195	0.016	
z-CFA_Left_ DD	Constant	−0.107	0.107		0.317	0.067
	z-BMI	0.170	0.069	0.196	0.007	
	Δz-BWH 0–12 m* z-pSBP	−0.130	0.048	−0.218	0.015	
Δz-BW (0–24 m) included as an independent variable						
z-CCA_Right_ IMT	Constant	−0.230	0.128		0.855	0.143
	Δz-BW 0–24 m	0.366	0.098	0.393	<0.001	
z-CCA_Left_SystD	Constant	−0.173	0.096		0.070	0.144
	z-BMI	0.228	0.062	0.305	<0.001	
	Δz-BW 0–24 m	0.136	0.064	0.177	0.035	
z-CCA_Left_ DD	Constant	−0.152	0.098		0.121	0.118
	z-BMI	0.267	0.061	0.353	<0.001	
z-CCA _Left_ IMT	Constant	0.000	0.100		0.998	0.051
	Δz-BW 0–24 m	0.230	0.080	0.241	0.005	
Δz-BWH (0–24 m) included as an independent variable						
z-CCA_Right_ IMT	Constant	−0.030	0.129		0.815	0.145
	Δz-BWH 0–24 m	0.368	0.098	0.395	0.001	
z-CFA_Right_SystD	Constant	−0.104	0.150		0.493	0.167
	z-BMI	0.400	0.108	0.409	0.001	
	z-BWH at birth	−0.258	0.111	−0.258	0.023	
z-CFA_Right_ DD	Constant	−0.181	0.144		0.213	0.213
	Δz-BWH 0–24 m	0.219	0.100	0.240	0.031	
	z-BMI	0.320	0.106	0.329	0.004	
	Δz-BWH 0–24 m* z-pSBP	−0.202	0.087	−0.251	0.024	
Δz-BW (12–24 m) included as an independent variable						
z-CCA_Left_SystD	Constant	0.168	0.095		0.081	0.120
	z-BMI	0.268	0.059	0.355	0.001	
z-CCA_Left_ DD	Constant	−0.151	0.097		0.122	0.118
	z-BMI	0.270	0.060	0.352	0.001	
z-CFA_Left_SystD	Constant	−0.118	0.114		0.299	0.036
	z-BMI	0.178	0.072	0.208	0.014	
z-CFA_Left_ DD	Constant	−0.082	0.113		0.469	0.025
	z-BMI	0.151	0.071	0.179	0.036	

βu and βs: non-standardized and standardized coefficients, respectively. Adj R^2^: adjusted R^2^ coefficient. SE: standard error.z: z-score. BW, BH, BWH: body weight, body height and BW for BH, respectively. BMI: body mass index. Δ: change in the analyzed period. SBP, DBP, PP and MBP: systolic, diastolic, pulse and mean blood pressure, respectively (p: peripheral, c: central). SVR: systemic vascular resistance. AIx, AIx@75: aortic augmentation index without and with heart rate correction (75 beats/minute). AP: augmented pressure. Pf, Pb: forward and backward wave component, respectively. DD, SystD: diastolic and systolic diameter, respectively. CCA, CFA: common carotid and common femoral artery, respectively. EM: elastic modulus. IMT: intima–media thickness. cfPWV: carotid–femoral pulse wave velocity. CI: cardiac index. A p < 0.05 was considered statistically significant. Variables entered in the model (forward method): Δz-BW 12–24 m, z-BMI, z-BWH at birth, hypertension (HT; 0 = no, 1 = yes), z-pSBP, interaction between anthropometric indexes and current z-BMI, z-pSBP and z-BWH at birth, marked as “*”. Only significant (*p <* 0.05) independent variables are shown.

**Table 7:** MLR analysis between arterial parameter z-scores at age 6 y (dependent variables) and anthropometric measures and CRFs (independent variables).

Dependent Variable	Independent Variables	βu	SE	βs	P	Adj R^2^
Δz-BW (0–36 m): included as an independent variable						
z-pSBP	Constant	−0.125	0.094		0.189	0.127
	z-BMI	0.201	0.065	0.227	0.020	
	Hypertension	0.944	0.359	0.177	0.009	
	Δz-BW 0–36 m	0.169	0.068	0.183	0.013	
z-cSBP	Constant	−0.130	0.090		0.150	0.078
	z-BMI	0.216	0.056	0.272	<0.001	
	Hypertension	0.712	0.333	0.151	0.034	
z-cPP	Constant	0.041	0.075		0.586	0.021
	Δz-BW 0–36 m* z-pSBP	0.101	0.045	0.161	0.027	
z-Pf	Constant	0.052	0.077		0.500	0.048
	Δz-BW 0–36 m	0.198	0.062	0.230	0.020	
z-CI	Constant	0.082	0.181		0.666	0.680
	Δz-BW 0–36 m* z-pSBP	−0.444	0.111	−0.852	0.070	
z-CCA_Right_ IMT	Constant	−0.026	0.122		0.834	0.089
	Δz-BW 0–36 m	0.312	0.103	0.315	0.030	
z-CCA_Left_ SystD	Constant	−0.125	0.093		0.178	0.112
	z-BMI	0.261	0.060	0.344	<0.001	
z-CCA_Left_ IMT	Constant	−0.003	0.096		0.976	0.047
	Δz-BW 0–36 m	0.227	0.080	0.231	0.050	
Δz-BW (12–36 m): included as an independent variable						
z-cSBP	Constant	−0.070	0.052		0.887	0.512
	z-pSBP	0.641	0.045	0.717	<0.001	
z-AIx	Constant	−0.124	0.070		0.078	0.124
	z-pSBP	−0.299	0.060	0.336	<0.001	
	Δz-BW 12–36 m* z-BWH at birth	−0.109	0.051	−0.145	0.033	
z-AIx@75	Constant	−0.119	−0.074		0.108	0.109
	z-pSBP	−0.242	−0.063	−0.271	<0.001	
	Δz-BW 12–36 m	−0.169	−0.068	−0.176	0.013	
z-AP	Constant	−0.130	0.073		0.076	0.087
	z-pSBP	−0.224	0.062	−0.247	<0.001	
	Δz-BW 12–36 m* z-BWH at birth	−0.146	0.053	−0.191	0.060	
z-Pf	Constant	0.135	0.072		0.062	0.188
	z-pSBP	0.353	0.060	0.391	<0.001	
	Δz-BW 12–36 m	0.155	0.065	0.158	0.018	
z-CI	Constant	−0.702	0.224		0.017	0.451
	Δz-BW 12–36 m	−0.579	0.210	−0.721	0.028	
z-CCA _Right_ SystD	Constant	−0.111	0.113		0.328	0.195
	z-BMI	0.314	0.079	0.399	<0.001	
	Δz-BW 12–36 m	0.260	0.091	0.277	0.005	
	z-pSBP	−0.175	0.081	−0.215	0.035	
z-CCA_Right_DD	Constant	−0.099	0.115		0.393	0.174
	z-BMI	0.305	0.080	0.387	<0.001	
	Δz-BW 12–36 m	0.236	0.092	0.250	0.012	
	z-pSBP	−0.175	0.082	−0.215	0.037	
z-CCA_Right_ IMT	Constant	0.022	0.110		0.842	0.197
	Δz-BW 12–36 m* z-pSBP	−0.409	0.085	−0.565	<0.001	
	z-pSBP	−0.276	0.118	−0.274	0.022	
z-CCA_Left_ SystD	Constant	−0.13	0.09		0.160	0.113
	z-BMI	0.26	0.06	0.35	<0.001	
z-CCA_Left_ DD	Constant	−0.114	0.094		0.227	0.110
	z-BMI	0.262	0.060	0.341	<0.001	
z-CFA_Left_ SystD	Constant	0.111	0.089		0.213	0.067
	Δz-BW 12–36 m	0.285	0.085	0.271	0.010	
z-CFA_Left_ DD	Constant	0.107	0.088		0.225	0.048
	Δz-BW 12–36 m	0.242	0.085	0.234	0.005	
Δz-BW (24–36 m): included as an independent variable						
z-cPP	Constant	0.052	0.067		0.441	0.121
	z-pSBP	0.296	0.057	0.354	0.001	
z-AIx@75	Constant	−0.098	0.070		0.165	0.126
	z-pSBP	−0.306	0.060	−0.346	0.001	
	Δz-BW 24–36 m* z-BWH at birth	−0.202	0.097	−0.141	0.040	
z-Pf	Constant	0.089	0.068		0.192	0.186
	z-pSBP	0.362	0.059	0.408	0.001	
	Δz-BW 24–36 m	0.233	0.106	0.145	0.030	
z-CCA_Right_ DD	Constant	−0.087	0.122		0.481	0.129
	z-BMI	0.223	0.083	0.283	0.009	
	Δz-BW 24–36 m	0.337	0.158	0.224	0.036	
z-CCA_Right_ IMT	Constant	−0.028	0.125		0.821	0.083
	Δz-BW 24–36 m* z-pSBP	−0.394	0.136	−0.308	0.005	

Abbreviations: identical to Table 6.

## Data Availability

The data presented in the study are available within the article and in Supplementary Material.

## Supplementary Materials

The following are available online at https://www.mdpi.com/2308-3425/8/2/20/s1. Table S1. Clinical, anthropometric, hemodynamic and arterial data for the reference population.

## Abbreviations

AIx	central (aortic) augmentation index
AIx@75	AIx adjusted to a 75 beats/min heart rate
AP	central (aortic) augmented pressure
BH	body height
BMI	body mass index
BW	body weight
BWH	body weight for body height
cBP	central (aortic) blood pressure
CCA	common carotid artery
CFA	common femoral artery
cfPWV	carotid–femoral pulse wave velocity
CRFs	cardiovascular risk factors
CV	cardiovascular
CVD	cardiovascular disease
DD	diastolic arterial diameter
EM	pressure-strain arterial elastic modulus
HBP	high blood pressure levels
HR	heart rate
IMT	intima–media thickness
MLR	multiple linear regression
m	months
MV	mean value
Pb	amplitude of the cBP backward component
pBP	peripheral (brachial) blood pressure
pDBP	peripheral (brachial) diastolic blood pressure
Pf	amplitude of the cBP forward component
pMBP	peripheral (brachial) mean blood pressure
pPP	peripheral (brachial) pulse pressure
pSBP	peripheral (brachial) systolic blood pressure
PWA	pulse wave analysis
SystD	systolic arterial diameter
SD	standard deviation
Y	years
Z	z-score
