# Supplementary material for: Changes in Body Size during Early Growth Are Independently Associated with Arterial Properties in Early Childhood
Source: J Cardiovasc Dev Dis. 2021 Feb 17;8(2):20. doi: 10.3390/jcdd8020020 (PMC7921917; doi:10.3390/jcdd8020020)
Supplement: Supplementary file 1 [file jcdd-08-00020-s001.pdf]

**Table Supplementary 1: Clinical, anthropometric and arterial haemodynamic, structural and functional parameters of reference population (N=400; Females: 46%)**							
	**MV**	**SD**	**Min.**	**p25th**	**p50th**	**p75th**	**Max.**
Clinical and anthropometric characteristics							
Age (years)	**6.0**	0.3	5.2	5.8	6.0	6.2	6.6
BW (kg) at 6 y	**20.8**	2.6	15.5	19.0	20.6	22.6	32.4
BH (m) at 6 y	**1.13**	0.05	1.01	1.10	1.13	1.16	1.29
BMI (Kg/m^2) at 6 y	**16.17**	1.28	12.27	15.25	16.11	16.96	22.15
z-BMI (SD) at 6 y	**0.51**	0.79	-2.00	-0.01	0.55	1.06	2.00
z-BW for age at 6 y (SD)	**0.07**	0.82	-2.08	-0.54	0.09	0.66	2.43
z-BH for age at 6 y (SD)	**-0.46**	0.91	-3.09	-1.11	-0.47	0.10	2.57
Obesity (n, %)				**0 (0)**			
Dyslipidemia (n, %)				**0 (0)**			
Diabetes (n, %)				**0 (0)**			
Hypertension (n, %)				**0 (0)**			
CVD Family background (n, %)				**0 (0)**			
Current Smoke (n, %)				**0 (0)**			
z-BW for age at birth (SD)	0.08	0.82	-1.85	-0.54	0.07	0.68	2.95
z-BW for age at birth (SD)	-0.23	1.05	-4.91	-0.88	-0.08	0.46	2.70
z-BMI for age at birth (SD)	0.33	0.99	-2.20	-0.33	0.41	0.93	3.65
z-BW for BH at 6 m. (SD)	0.38	1.04	-3.40	-0.25	0.40	1.01	4.89
z-BW for age 6 m. (SD)	0.02	0.86	-2.14	-0.57	-0.03	0.64	2.74
z-BH for age 6 m. (SD)	-0.30	1.15	-3.73	-1.02	-0.47	0.32	5.19
z-BMI for age 6 m. (SD)	0.27	1.05	-3.89	-0.36	0.29	0.91	4.52
z-BW for BH 12 m. (SD)	0.23	0.94	-2.47	-0.40	0.23	0.80	3.80
z-BW for age 12 m. (SD)	0.50	1.05	-2.72	-0.17	0.47	1.20	5.51
z-BH for age 12 m. (SD)	-0.30	1.14	-4.72	-1.01	-0.41	0.37	5.57
z-BMI for age 12 m. (SD)	0.55	1.09	-2.70	-0.15	0.51	1.26	5.84
z-BW for BH 24 m. (SD)	0.40	1.08	-3.86	-0.23	0.34	1.05	4.59
z-BW for age 24 m. (SD)	0.20	0.94	-2.83	-0.48	0.20	0.79	3.52
z-BH for age 24 m. (SD)	-0.22	1.06	-4.12	-0.95	-0.27	0.52	2.55
z-BMI for age 24 m. (SD)	0.48	1.13	-4.07	-0.21	0.41	1.17	4.72
z-BW for BH 36 m. (SD)	0.32	0.96	-2.91	-0.30	0.31	0.90	4.41
z-BW for age 36 m. (SD)	0.11	0.90	-2.32	-0.50	0.11	0.73	2.81
z-BH for age 36 m. (SD)	-0.20	1.06	-3.18	-0.95	-0.27	0.48	2.93
z-BMI for age 36 m. (SD)	0.33	0.99	-3.05	-0.28	0.28	0.99	4.57
Central and peripheral haemodynamics parameters							
Heart rate (beats/minute)	90	11	66	83	89	98	128
pSBP (mmHg)	99	7	80	93	98	103	120
pDBP (mmHg)	58	6	45	54	58	62	74
pPP (mmHg)	40	7	26	36	40	45	60
pMBP (mmHg)	72	6	57	67	71	75	86

cSBP (mmHg)	82	6	64	78	82	86	100
cDBP (mmHg)	60	6	46	56	60	64	75
cPP (mmHg)	22	5	7	19	21	25	43
CO (liter/minute)	4.5	1.2	3.7	4	4.4	4.7	5.3
CI (liter/minute/m^2)	4.8	0.3	4.2	4.3	4.9	5.2	5.4
SVR (s.mmHg/ml)	1.10	0.07	0.92	1.04	1.09	1.14	1.24
AIx (%)	10	10	-16	4	11	17	37
AIx@75 (%)	17	10	-10	11	17	24	43
AP (mmHg)	2	2	-5	1	2	4	9
Pf (mmHg)	20	5	7	17	19	23	43
Pb (mmHg)	10	5	3	9	10	11	78
Structural arterial parameters							
Right CCA SystD (mm)	6.04	0.57	4.83	5.63	5.98	6.45	7.41
Right CCA DD (mm)	5.40	0.53	4.21	5.02	5.35	5.82	6.91
Right CCA IMT (mm)	0.422	0.026	0.370	0.409	0.420	0.431	0.496
Left CCA SystD (mm)	5.87	0.48	4.84	5.54	5.84	6.19	7.48
Left CCA DD (mm)	5.21	0.44	4.25	4.91	5.18	5.48	6.99
Left CCA IMT (mm)	0.419	0.026	0.356	0.404	0.415	0.430	0.567
Right CFA SystD (mm)	4.75	0.48	3.49	4.40	4.74	5.00	6.50
Right CFA DD (mm)	4.43	0.47	3.39	4.11	4.42	4.69	6.11
Right CFA IMT (mm)	0.330	0.029	0.285	0.313	0.323	0.334	0.411
Left CFA SystD (mm)	4.74	0.48	3.74	4.37	4.70	5.06	6.35
Left CFA DD (mm)	4.43	0.49	3.41	4.09	4.41	4.76	5.98
Left CFA IMT (mm)	0.335	0.027	0.271	0.318	0.331	0.352	0.398
Functional arterial parameters (Local and regional arterial stiffness)							
Right CCA EM (mmHg)	186.3	46.7	71.4	151.8	188.8	216.5	304.0
Left CCA EM (mmHg)	178.3	49.4	54.1	142.8	172.6	202.9	313.8
Right CFA EM (mmHg)	637.8	297.7	276.6	416.1	574.8	724.9	1554.8
Left CFA EM (mmHg)	596.1	229.8	218.9	419.4	544.6	727.1	1561.3
cfPWV (m/s)	4.8	0.7	2.9	4.4	4.7	5.2	7.7

MV: mean value. SD: standard deviation. Min. and Max.: Minimal and maximal value. z: z-score. p25th, p50th and p75th: percentile 25, 50 (median) and 75. BMI: body mass index. CV: cardiovascular. CVD: cardiovascular disease. CO: cardiac output. CI: cardiac index. pSBP, pDBP, pPP and pMBP: peripheral (brachial) systolic, diastolic, pulse and mean blood pressure. cSBP, cDBP, and cPP: central (aortic) systolic, diastolic, and pulse pressure. SVR: systemic vascular resistances. AIx and AIx@75: aortic augmentation index without and with normalization at 75 beats/minute heart rate. AP: central (aortic) augmented pressure. Pf and Pb: forward and backward aortic blood pressure component. CCA and CFA: common carotid and femoral artery. EM: pressure-strain elastic modulus. IMT: intima-media thickness. cfPWV: carotid-femoral pulse wave velocity. BW: Body weight. BH: Body height. BMI: body mass index.
